# YOLO-WSR: A wavelet feature and structure guided diffusion model for detection and automated restoration of cultural heritage artifact images

**DOI:** 10.1371/journal.pone.0351571

**Published:** 2026-09-11

**Authors:** Yi Chen, Xing Wang, Wang Shaohua

**Affiliations:** 1 Academy of Fine Arts and Crafts, Zhejiang Guangsha Vocational and Technical University of Construction, Dongyang, China; 2 Zhejiang Guangsha Vocational and Technical University of Construction, Dongyang, China; 3 Advanced Institute of Information Technology Peking University, Dongyang, China; National Taichung University of Science and Technology, TAIWAN

## Abstract

In response to the complexity and diversity involved in damage detection and automated restoration of cultural heritage artifact images associated with intangible cultural heritage preservation, this study proposes a YOLO-based restoration framework that integrates wavelet features with structure-guided diffusion, termed YOLO-WSR. The proposed method enhances detection accuracy through wavelet feature upsampling and employs a structure-guided diffusion generation mechanism to achieve effective restoration of texture and structural details in damaged artifact images. In addition, a CLIP-IQA-based feedback optimization module is introduced to perform no-reference quality assessment on the restored outputs and iteratively refine the restoration results, thereby forming a closed-loop collaborative framework that integrates detection, restoration, and evaluation. Experiments conducted on multiple publicly available datasets demonstrate the effectiveness of YOLO-WSR. The results show that the proposed method consistently improves performance over existing approaches, achieving approximately 5–6% improvement in mAP@50 for damage detection, while obtaining enhanced PSNR, SSIM, LPIPS, and SRCC performance for restoration quality evaluation. Further ablation studies demonstrate the contribution of each component to the overall framework. The proposed method provides an effective and quantifiable technical solution for the digital preservation of cultural heritage artifact images, offering practical support for accurate damage identification, automated restoration, and intelligent management of visual cultural heritage resources.

## Introduction

As important carriers of historical and cultural memory, cultural heritage artifacts related to intangible cultural heritage preservation embody rich artistic, craftsmanship-related, and historical information. However, under the long-term influence of aging, environmental conditions, and human activities, these artifacts and their digital visual records are often affected by cracks, fading, missing regions, and other forms of deterioration, posing substantial challenges to cultural heritage preservation and digital reconstruction [1]. Meanwhile, with the rapid development of heritage digitization and digital museum exhibitions, high-precision inspection and automated restoration of cultural heritage artifact images using computer vision and artificial intelligence have become increasingly important in both research and practical applications [2,3]. Conventional manual restoration is not only time-consuming and labor-intensive, but also difficult to standardize in terms of consistency and reproducibility. Therefore, investigating image-analysis-based techniques for cultural heritage artifact image damage detection and automated restoration is of considerable academic significance and practical value [4].

In the fields of artifact damage detection and image restoration, a range of methods have achieved notable progress. For instance, convolutional neural network (CNN)-based texture classification methods have been applied to identify subtle cracks, faded regions, and local pattern losses on artifact surfaces by extracting discriminative local texture representations through hierarchical convolutional layers [5]. Multi-scale feature pyramid networks (FPNs) improve the detection of small targets, fine-grained details, and damaged regions under complex backgrounds by integrating feature maps from different semantic levels [6]. Deep residual networks (ResNets), when combined with self-attention mechanisms, can further emphasize surface details of artifacts and alleviate the problem of insufficient local texture contrast [7]. Graph neural networks (GNNs) have also been introduced to model structural relationships in cultural relic images, enabling the analysis of spatial dependencies and structural correlations among damaged regions, which is beneficial for maintaining the continuity of restored local textures [8]. In addition, generative adversarial network (GAN)-based restoration methods can provide preliminary texture completion for missing areas, where adversarial training between the generator and discriminator helps produce visually plausible local texture patterns. Meanwhile, multi-channel attention convolutional networks and dilated convolutional networks have been used for fine crack detection and edge enhancement in artifact images, with dilated convolutions improving sensitivity to sparse cracks and small-scale damage [9]. Although these approaches have addressed certain issues in cultural relic image processing to some extent, several challenges remain, including insufficient detection accuracy, incomplete preservation of texture details, limited structural continuity, and the lack of quantitative evaluation for restoration quality. Although these approaches have addressed certain issues in cultural relic image processing to some extent, several challenges remain, including insufficient detection accuracy, incomplete preservation of texture details, limited structural continuity, and the lack of quantitative evaluation for restoration quality. These limitations become more evident in cultural heritage artifact images associated with intangible cultural heritage preservation, which often contain complex backgrounds and densely distributed local damage.

In recent years, with the rapid development of deep learning, various advanced methods have shown considerable potential in artifact image restoration and digital reconstruction. For missing-region generation, variational autoencoders (VAEs) learn latent representations of artifact images and reconstruct damaged regions through reparameterization, thereby enabling effective recovery of local texture structures [10]. Diffusion models generate high-fidelity textures through a progressive denoising process, leading to clear improvements in the continuity and naturalness of restored regions, particularly for complex cracks and localized surface wear [11]. To enrich feature representation, cross-modal feature fusion methods integrate image features with multi-source information, such as texture templates, depth cues, and color channels, allowing more fine-grained characterization of damaged areas [12]. Multi-scale super-resolution reconstruction techniques can recover details and hierarchical texture information from low-resolution images, thereby improving the recognizability of artifact patterns and the clarity of microscopic structures. To maintain stylistic consistency in restoration results, reference-guided restoration methods employ reference images from artifacts of the same type or with similar decorative patterns to guide texture generation in missing regions, making the completed content more consistent with the original artifact style [13]. In addition, local structure-constrained networks and boundary-preserving convolutional methods play important roles in texture reconstruction and missing-region completion, as they help preserve edge continuity and detail fidelity in restored areas [14]. Although these methods perform well in missing-content generation and texture-structure recovery, they still have limitations in fine-grained boundary refinement, iterative automated restoration, and no-reference quality assessment, making it difficult for them to fully meet the requirements of complete artifact restoration and high-quality digital reconstruction of cultural heritage visual resources associated with intangible cultural heritage preservation.

To address the aforementioned issues, including insufficient detection accuracy, discontinuities in texture and structural information, and the difficulty of adaptively evaluating restoration quality, this study proposes a detection-driven automated restoration system for cultural heritage artifact images, termed YOLO-WSR (YOLO-based Wavelet-feature and Structure-guided Restoration). Different from conventional pipelines that independently perform damage detection, image restoration, and quality assessment, YOLO-WSR constructs a collaborative closed-loop framework by establishing information interaction and feedback optimization among these three processes. The proposed system consists of three interconnected components. First, the artifact damage detection module based on wavelet-enhanced YOLO performs accurate localization of cracks, fading, missing regions, and fine decorative patterns, while the generated damage masks and multi-scale feature representations are further transferred to the restoration stage as spatial and texture priors. This detection-guided interaction enables the restoration process to focus on damaged regions and preserve artifact-specific structural details rather than performing unconstrained image generation. Second, the automated restoration generation module introduces damage-aware structural constraints into the diffusion-based restoration process, where detection-derived masks and local structural information guide progressive generation to improve texture reconstruction and structural continuity in complex damaged areas. Third, the restoration quality assessment and feedback optimization module evaluates restoration results through CLIP-based quality estimation and provides adaptive feedback to refine subsequent restoration procedures, thereby transforming the restoration process from one-time generation into iterative quality-aware optimization. Overall, the main contribution of YOLO-WSR lies in the cross-task interaction and feedback-driven optimization strategy that connects detection, generation, and evaluation, rather than simply integrating existing models. By enabling mutual information exchange among different stages, the proposed framework provides an effective approach for intelligent cultural heritage artifact image restoration and demonstrates improved performance under the evaluated experimental settings. The specific contributions of this study are as follows:

- A wavelet feature enhancement-based strategy is proposed for artifact damage detection, which improves the recognition accuracy of cracks, missing regions, and decorative pattern areas.
- A structure-guided diffusion restoration module is designed to achieve reasonable completion of both texture and structural information in damaged regions.
- A no-reference quality assessment and iterative feedback mechanism is introduced, providing a quality control and optimization pathway for automated restoration and ensuring the reliability and practical usability of the final restoration results.

The remainder of this paper is organized as follows. Section 2 reviews related studies on artifact image detection and restoration, covering damage detection, image restoration, feature enhancement, generative modeling, and quality evaluation. Section 3 presents the proposed YOLO-WSR model in detail, including its overall architecture and the design principles of each module. Section 4 describes the experimental datasets, implementation settings, evaluation metrics, and comparative experiments, followed by a detailed analysis of model performance and ablation results. Finally, Section 5 concludes the paper by summarizing the main findings and outlining potential directions for future research.

## Related work

### Deep learning-based damage detection for cultural heritage artifacts

In recent years, with the continuous advancement of computer vision and artificial intelligence, deep learning has been widely applied to damage detection in cultural heritage artifacts [15]. It enables accurate identification of subtle defects, such as cracks, fading, and missing regions, even under complex background conditions, thereby providing technical support for digital restoration and cultural heritage preservation. DenseNet-based feature fusion networks employ dense connections to facilitate the transmission and reuse of multi-level features, which improves sensitivity to fine cracks and faded areas while enhancing the representation of local details [16]. Despite these advantages, their complex network structures lead to high computational costs and relatively slow inference, making them less suitable for real-time applications. Improved U-Net++ segmentation networks integrate multi-scale skip connections and deep feature fusion, which can significantly refine the boundaries of damaged regions and strengthen contextual representation. Nevertheless, when processing artifact images with rich textures or complex structures, such models may produce over-smoothed outputs, resulting in the loss of edge details in fine cracks and small missing areas [17]. Lightweight MobileNet-FPN detection networks improve the recognition of small targets and local textures while maintaining efficient inference speed, making them suitable for deployment in resource-constrained environments [18]. Despite their efficiency, their feature representation capacity remains limited, and missed detections may still occur for fine cracks in complex backgrounds or highly noisy images. Deformable convolutional networks can adaptively model the shapes of irregular damaged regions and improve the localization of complex cracks and irregular decorative patterns, but their training process is sensitive to hyperparameters and initial weights, which may lead to unstable convergence or overfitting [19]. Attention-guided residual networks assign higher weights to critical damaged regions through attention mechanisms, thereby improving the recognition accuracy of low-contrast regions and local details. Nevertheless, in areas with complex textures or densely distributed targets, attention mechanisms may overemphasize certain local features and thus affect the consistency of overall detection [20]. Although these methods have achieved certain improvements in damage detection accuracy and detail preservation, they still face limitations when dealing with intangible cultural heritage artifact images characterized by complex backgrounds, overlapping textures, or blurred boundaries [21].

Compared with the aforementioned methods, the proposed YOLO-WSR integrates feature enhancement strategies with a small-target localization mechanism in the artifact detection module. This design enables accurate detection of cracks, missing regions, and fine decorative patterns, while also providing high-quality damage masks for the subsequent automated restoration module. As a result, detection and restoration can be jointly optimized within a unified workflow, leading to improved performance in cultural heritage artifact image processing associated with intangible cultural heritage preservation.

### Image restoration techniques for heritage objects

In the field of artifact image restoration, the rapid development of artificial intelligence and the growing maturity of computer vision techniques have made deep learning an important tool for cultural heritage preservation and digital reconstruction. In recent years, various methods have achieved considerable progress in tasks such as crack detection, fading restoration, missing-region completion, and texture reconstruction [22]. Context Encoders complete missing regions by learning contextual information around damaged areas and can restore the global structure of images with relatively regular defects. Despite these advantages, in regions with complex textures or rich details, they tend to generate blurred or visually unnatural patterns, which weakens the realism of microscopic details. EdgeConnect first employs an edge prediction network to recover structural contours in missing regions and then performs texture completion, showing advantages in maintaining global structural consistency [23]. Nevertheless, for artifact images with complicated boundaries, severely broken cracks, or large missing areas, its structural prediction may still be inaccurate, which further affects the natural transition of restored textures. Partial Convolution Networks avoid interference from invalid pixels by performing convolution only on valid regions, thereby improving the restoration of partially damaged areas. Despite their effectiveness, when dealing with large missing regions or continuous texture patterns, they may produce texture breaks and discontinuities, reducing the overall coherence of the restored image [24]. Generative Multi-column Networks use multi-branch generators to process features at different scales simultaneously, allowing more details to be recovered in complex texture regions and enhancing image realism [25]. Even so, their complicated architecture leads to high computational cost and low inference efficiency, making them less suitable for real-time or large-scale restoration scenarios. Texture Synthesis Networks complete damaged regions by generating similar texture patches and can achieve favorable results in areas with repeated motifs or regular patterns [26]. Nevertheless, for artifact images containing non-repetitive, complex, or structurally diverse textures, they may introduce local texture inconsistency or stylistic mismatch with surrounding regions. Overall, these methods can recover texture information and global structures in damaged regions to a certain extent, providing feasible solutions for preliminary artifact image restoration [27]. Yet, they still show clear limitations in handling complex decorative patterns, hierarchical textures, subtle cracks, and local structural continuity in cultural heritage images.

The proposed YOLO-WSR performs texture completion and structural reconstruction in the automated restoration generation module by jointly utilizing damage masks and local structural information. This design not only preserves the continuity of cracks and missing regions, but also introduces quality feedback and iterative optimization into the restoration process. As a result, the restored images achieve better texture naturalness and structural consistency than those produced by conventional methods. Moreover, YOLO-WSR enables collaborative optimization between damage detection and image restoration, thereby improving the overall reliability and effectiveness of automated artifact restoration.

### Perceptual quality assessment of restored cultural images

In the field of image quality assessment, particularly for no-reference evaluation of restored cultural heritage images, the rapid development of deep learning and computer vision has led to the emergence of various deep neural network-based methods for automatically assessing overall image quality, local texture details, and the visual authenticity of restoration results. BRISQUE-Net learns natural scene statistical features to perform no-reference quality scoring and can effectively evaluate brightness, contrast, and texture uniformity. It performs well on images with relatively simple textures and obvious illumination variations; nevertheless, its sensitivity and accuracy remain limited when applied to artifact images containing complex textures and rich hierarchical structural information [28]. NIQE-based CNN methods combine unnatural image statistical features with convolutional networks to predict image quality, enabling the monitoring and quantification of global quality variations. Nevertheless, for restored images with abundant local details or small damaged regions, their evaluation results may be biased and may fail to accurately reflect local restoration quality [29]. DeepBIQ extracts image features through multi-scale convolution and conducts comprehensive quality evaluation, showing sensitivity to changes in brightness, contrast, noise level, and overall texture continuity. It is relatively effective for artifact images with uneven illumination and color distribution, but it may still overlook subtle local variations in regions with complex textures, cracks, or minor defects [30]. RankIQA adopts contrastive learning to rank and score images, allowing the model to learn relative quality relationships from large-scale datasets and improving its stability and generalization through ranking-based training. Nevertheless, due to the lack of explicit absolute quality quantification, its evaluation outputs are difficult to use directly for guiding restoration strategies in terms of specific numerical criteria [31]. The Multi-scale Ensemble Opinion Network (MEON) approximates subjective perceptual evaluation by integrating features at multiple scales and can achieve relatively high accuracy in overall image assessment. Nevertheless, in regions with complex textures, small-scale local damage, or fine cracks, its ability to judge local restoration quality remains limited [32]. Although these methods have achieved certain progress in global image quality prediction, they are still insufficient for no-reference assessment of restored cultural heritage images, especially in evaluating fine cracks, texture continuity in locally missing regions, and detail fidelity [33]. As a result, they cannot fully and accurately characterize the true quality of artifact restoration outcomes.

YOLO-WSR incorporates a restoration quality assessment and feedback optimization module in which a visual-semantic embedding model is employed to quantitatively score the restored results, while the damage-region masks are jointly used to analyze local texture fidelity and structural continuity. This design not only enables a more refined no-reference evaluation of restoration quality, but also allows the assessment outcomes to be fed back into the automated restoration module for iterative optimization. In this way, YOLO-WSR establishes a closed-loop collaborative framework that integrates detection, restoration, and quality evaluation, thereby significantly improving the stability and reliability of the final restoration results.

## Model

### Ethics statement

This study strictly adheres to ethical standards in research and publication. All data used in this work are obtained from publicly available datasets with open access, ensuring no privacy, copyright, or confidentiality violations. No human participants, sensitive personal information, or animal subjects were involved in the experiments. The methods proposed in this work are applied solely to digital images of cultural heritage artifacts for research purposes, aiming to improve automated detection and restoration.

### Overview of our model

**Fig 1:**
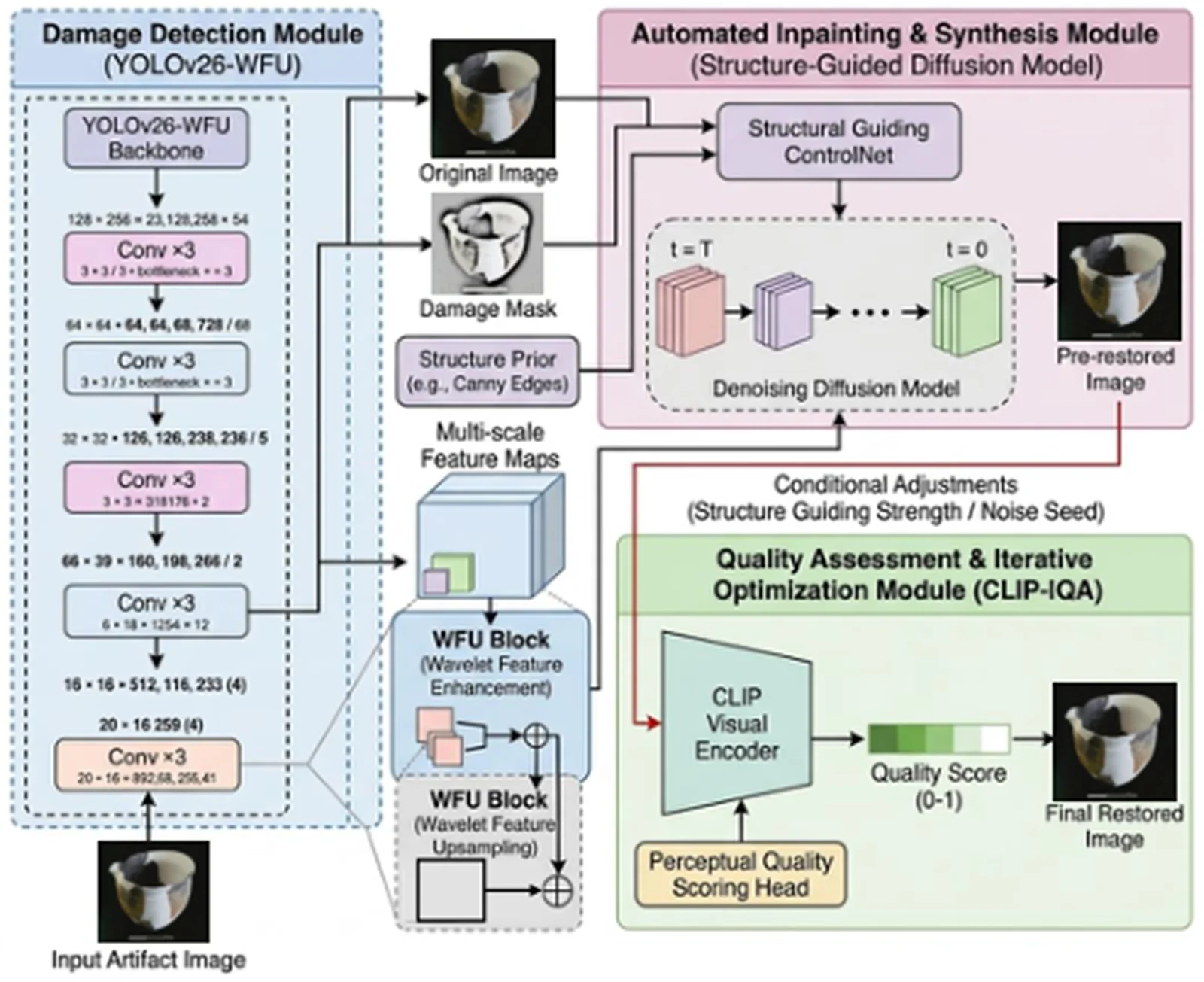
Overall architecture of YOLO-WSR framework and the closed-loop workflow for cultural heritage artifact damage detection, restoration, and quality feedback optimization. The research design and evaluation are based on the open-access CHVD, HBDD, and DUA datasets (see Data Availability Statement for URLs).

The proposed YOLO-WSR model is a detection and automated restoration system designed for intangible cultural heritage artifact images. Through the coordinated operation of three core modules, the model establishes a closed-loop processing workflow that spans damage detection, automated restoration, and restoration quality assessment. The input artifact image is first subjected to feature extraction and preliminary processing, ensuring that texture details and damage-related information can be accurately captured. The model design emphasizes the collaborative relationship among different modules, where the detection results directly provide high-precision damage masks for the restoration module and offer quantifiable feedback evidence for the quality assessment module. This design improves the overall performance and controllability of the system in artifact image processing. The overall architecture is illustrated in Fig 1.

The Wavelet Feature Upsampling Detection Module adopts a YOLOv26 network integrated with WFU (wavelet feature upsampling) and serves as the first stage of the proposed closed-loop restoration framework. Different from conventional detection modules that only provide localization results, this module establishes a direct interaction between damage perception and subsequent restoration by generating both damage-region masks and multi-scale feature representations. It is responsible for accurately localizing and segmenting cracks, fading regions, missing areas, and fine decorative patterns in artifact images. By enhancing feature representations in the wavelet domain and aggregating multi-scale information, the module improves the perception capability for subtle textures and local defects while maintaining spatial consistency between detection outputs and the original images. The generated masks and intermediate feature maps are further transferred to the restoration stage as spatial and texture priors, enabling the subsequent diffusion model to perform damage-aware reconstruction rather than unconstrained image generation.

The Structure-Guided Diffusion Inpainting Module is built upon a structure-guided diffusion model and serves as the generation component of the proposed framework. Instead of directly applying a general diffusion model for image synthesis, this module incorporates detection-derived damage masks and structural feature maps extracted from the original image, such as Canny edge information, as conditional guidance during the restoration process. These spatial and structural constraints guide the diffusion sampling trajectory, allowing the generated textures and structures to maintain consistency with surrounding intact regions. Through iterative denoising and structure-aware generation, the module progressively reconstructs missing content while preserving artifact-specific patterns and spatial continuity, thereby improving the reliability of restoration results in complex damage scenarios.

The CLIP-based Quality Regression and Feedback Module introduces a quality-aware optimization mechanism to establish adaptive interaction between restoration generation and quality evaluation. It adopts a CLIP-based no-reference image quality assessment network, namely CLIP-IQA, to evaluate the perceptual quality of the initially restored image and output a quality score ranging from 0 to 1. By optimizing a lightweight quality regression head, the module enables the CLIP visual encoder to capture restoration-related characteristics, including texture naturalness, structural continuity, and detail recovery. When the restoration quality does not meet the predefined criterion, the quality feedback signal is transmitted back to the generation module to adjust the restoration process, such as modifying structural guidance strength or refining sampling strategies. This feedback-driven mechanism transforms restoration from a single-pass generation process into an iterative optimization procedure.

Through the coordinated interaction of the three modules, YOLO-WSR establishes a complete closed-loop pipeline consisting of damage perception, structure-guided reconstruction, and quality-aware refinement. The framework does not simply combine existing detection, diffusion, and evaluation models; instead, it enables information exchange across different stages, where detection provides restoration priors, restoration generates quality-evaluated outputs, and quality feedback further guides optimization. This collaborative design improves the controllability and adaptability of cultural heritage artifact image restoration and provides an effective framework for automated digital preservation.

### WFU-YOLO for damage detection

**Fig 2:**
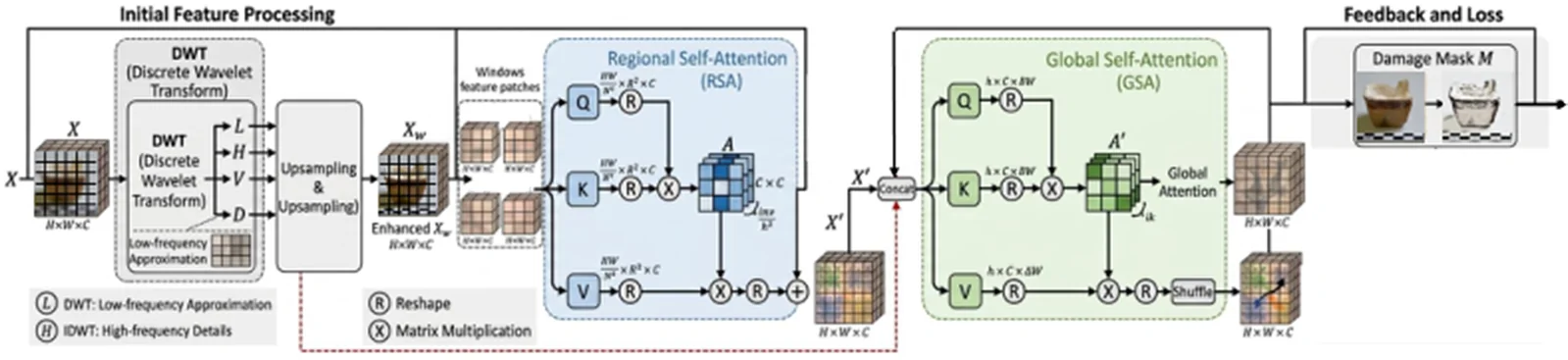
Detailed pipeline of the Wavelet Feature Upsampling Detection Module for damage localization and multi-scale feature enhancement. The research design and evaluation are based on the open-access CHVD, HBDD, and DUA datasets (see Data Availability Statement for URLs).

As the first functional component of the YOLO-WSR system, the Wavelet Feature Upsampling Detection Module is responsible for transforming raw artifact images into spatial priors and texture features that can be directly utilized by the subsequent restoration generation module. As illustrated in Fig 2, this module integrates WFU with the YOLOv26 network to achieve high-precision localization and instance segmentation of cracks, fading regions, missing areas, and fine decorative patterns, while simultaneously generating multi-scale feature maps to guide the subsequent automated restoration process. The core objective of this module is to ensure that damaged regions can be accurately captured under complex backgrounds and diverse material conditions, while preserving sufficient local texture information to provide a reliable data foundation for texture completion and structural reconstruction, thereby guaranteeing the accuracy and stability of the overall restoration pipeline.

The input feature map $F_{in}\in {\mathbb{R}}^{C\times H\times W}$ is first processed by the discrete wavelet transform (DWT) to obtain the low-frequency approximation component *L* and the high-frequency detail components, denoted as $\left\{L,H,V,D\}=DWT(F_{in}\right)$. Specifically, *L* preserves the global structural information, while *H*, *V*, and *D* retain texture details in the horizontal, vertical, and diagonal directions, respectively. Different from conventional interpolation-based upsampling in the feature pyramid, the proposed Wavelet Feature Upsampling (WFU) operation does not directly enlarge all wavelet sub-bands to the original feature resolution before reconstruction. Instead, DWT is introduced as a feature enhancement operation, where the decomposed wavelet components maintain compatible spatial dimensions during processing. The low-frequency and high-frequency components are enhanced separately to preserve complementary semantic and edge-related information, and inverse discrete wavelet transform (IDWT) is subsequently applied to reconstruct the enhanced feature representation. Therefore, WFU serves as a frequency-domain feature refinement strategy integrated into the feature fusion process rather than a replacement for the original upsampling operation. During the reconstruction process, the processed wavelet components are reconstructed through inverse discrete wavelet transform, generating the enhanced feature map $F_{out}$:


$$\begin{array}{c} F_{out}=IDWT(\hat{L},\hat{H},\hat{V},\hat{D}) \end{array}$$ (1)

The damage detection output of this module includes localization prediction, category prediction, and damage-region mask generation. The localization branch predicts bounding boxes of damaged regions, while the classification branch identifies the corresponding damage categories. Based on the detected bounding boxes, binary damage masks are further generated to indicate the spatial regions requiring restoration. Specifically, the pixels inside the detected damage bounding boxes are assigned as restoration regions, while the remaining pixels are retained as contextual background information. The generated binary masks establish the spatial correspondence between the detection module and the subsequent restoration module, enabling the diffusion-based restoration process to focus on damaged areas rather than performing unconstrained image generation.

For bounding box regression, the Complete Intersection over Union (CIoU) loss function is adopted, where IoU denotes the intersection over union between the predicted box *b* and the ground-truth box $b_{gt}$, $\rho^{2}(b,b_{gt})$ represents the squared Euclidean distance between the center points of the two boxes, $d_{enc}$ represents the diagonal distance of the minimum enclosing box, *v* measures the consistency of the aspect ratio, and α is the weighting coefficient. This loss function provides more stable regression for small crack regions and alleviates the gradient instability problem of conventional IoU loss when there is insufficient overlap:


$$\begin{array}{c} L_{CIoU}=1-IoU+\frac{\rho^{2}(b,b_{gt})}{d_{enc}^{2}}+\alpha v \end{array}$$ (2)

Category prediction is performed using Focal Loss, where *K* denotes the total number of categories, $p_{t,i}$ is the predicted probability assigned by the model to the ground-truth label of the *i*-th category, $\alpha_{i}$ is the class-balancing weight, and γ=2 is the focusing parameter. This design alleviates foreground-background imbalance and improves the classification capability for subtle damage samples. The balancing coefficients $\lambda_{box}=0.05$ and $\lambda_{cls}=0.5$ are introduced to ensure appropriate optimization weights between localization and classification:


$$\begin{array}{c} L_{Focal}=-\sum_{i=1}^{K}\alpha_{i}(1-p_{t,i}{)}^{\gamma }\log(p_{t,i}) \end{array}$$ (3)

$$\begin{array}{c} L_{det}=\lambda_{box}L_{CIoU}+\lambda_{cls}L_{Focal} \end{array}$$ (4)

After wavelet feature upsampling enhancement and YOLOv26-based feature extraction, the module generates multi-scale feature representations $F_{out}$. The localization and classification branches subsequently produce damage detection results, from which the corresponding binary damage mask *M* and fused multi-scale feature map $F_{fusion}$ are obtained. The generated mask *M* provides spatial guidance for the automated restoration generation module by defining the damaged regions to be reconstructed, while $F_{fusion}$ provides multi-level texture and semantic priors. These outputs are jointly transmitted to the subsequent restoration stage, constraining the restoration region and guiding structure-aware texture reconstruction. Therefore, the proposed detection module establishes an effective connection between damage localization and automated restoration.

### Structure-guided diffusion model for inpainting

**Fig 3:**
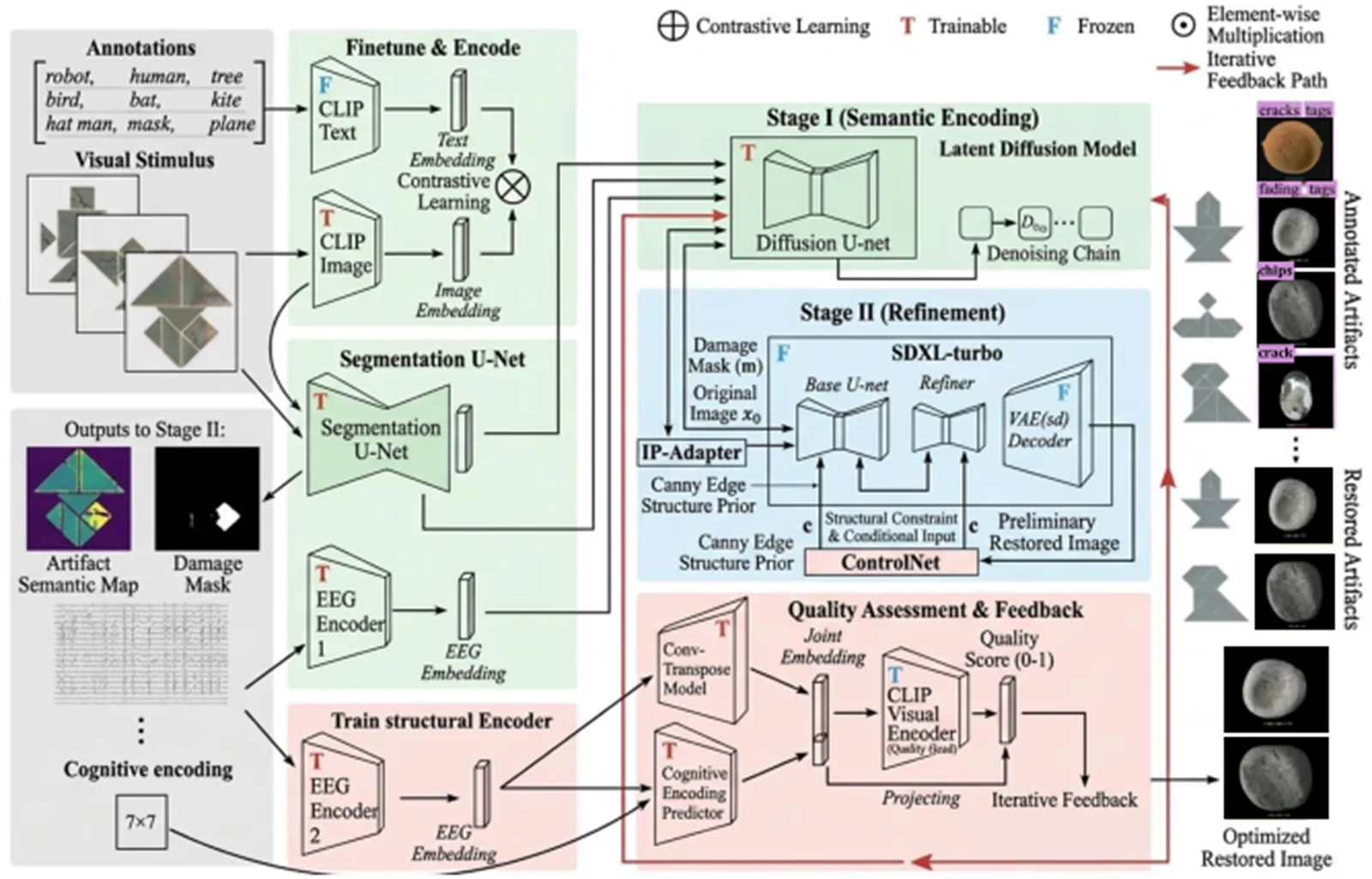
Structure-guided diffusion inpainting process using damage masks and structural feature guidance for artifact image restoration. The research design and evaluation are based on the open-access CHVD, HBDD, and DUA datasets (see Data Availability Statement for URLs).

The automated restoration generation module is built upon a structure-guided diffusion model. It takes the original artifact image, the damage mask produced by the artifact target detection module, and the structural feature map as joint conditional inputs to automatically restore cracks, missing regions, and locally damaged decorative patterns. The core objective of this module is to achieve high-fidelity completion of damaged areas while preserving the original texture and structural continuity of the artifact, and to prevent the generated results from deviating from the geometric contours and pattern boundaries of the original artifact through structural constraints. The entire module consists of three components: structural feature extraction, conditional diffusion generation, and structure-guided sampling. Through iterative denoising, the missing content is progressively recovered, and an initial restored image is finally produced. The overall architecture is illustrated in Fig 3.

In the forward process of the diffusion model, the system constructs a Markov chain that gradually degrades a real image into Gaussian noise. Let the diffusion time step be t=1,...,T, where the total number of steps is *T* = 1000. The parameter $\beta_{t}\in (0,1)$ denotes the predefined noise variance schedule and is set using a cosine scheduling strategy. Here, *I* denotes the identity matrix. After the continuous addition of noise, the original image is progressively degraded into a pure noise distribution, i.e., $x_{T}\sim 𝒩(0,I)$. In addition, $\alpha_{t}=1-\beta_{t}$, and ${\bar{\alpha }}_{t}=\prod_{i=1}^{t}\alpha_{i}$. The variable ϵ represents standard Gaussian noise with the same size as the original image. By progressively destroying the image structure, this process provides training samples for subsequent reverse denoising learning:


$$\begin{array}{c} p(x_{t}|x_{t-1})=𝒩(x_{t};\sqrt{1-\beta_{t}}x_{t-1},\beta_{t}I) \end{array}$$ (5)

$$\begin{array}{c} x_{t}=\sqrt{{\bar{\alpha }}_{t}}x_{0}+\sqrt{1-{\bar{\alpha }}_{t}}\epsilon ,\;\epsilon \sim 𝒩(0,I) \end{array}$$ (6)

In the reverse restoration stage, the conditional denoising network $\epsilon_{\theta }(x_{t},t,c)$ takes the current noisy image $x_{t}$, the time step *t*, and the conditional information *c* as inputs to predict the noise and progressively recover *t*he textures in the missing regions. Its training objective is to minimize the mean squared error between the predicted noise and the true noise, where 𝔼 denotes the mathematical expectation over the data distribution, time steps, and noise. Here, $\epsilon_{\theta }(\cdot )$ represents a noise prediction network based on a U-Net backbone with parameters θ. During training, the model mainly learns noise prediction over the regions specified by the damage mask, enabling the network to restore missing content under the constraint of structural conditions:


$$\begin{array}{c} L_{diff}={𝔼}_{x_{0},\epsilon ,t,c_{str}}[\|\epsilon -\epsilon_{\theta }(x_{t},t,c_{str}){\|}_{2}^{2}] \end{array}$$ (7)

To further enhance the conformity of the restoration results to the structural information of artifacts, this study introduces a structure-guided sampling mechanism during the sampling stage. Specifically, the system performs linear interpolation between the conditional and unconditional noise prediction results to obtain the refined noise estimate:


$$\begin{array}{c} {\tilde{\epsilon }}_{\theta }(x_{t},t,c_{str})=\epsilon_{\theta }(x_{t},t,\emptyset )+\omega_{str}(\epsilon_{\theta }(x_{t},t,c_{str})-\epsilon_{\theta }(x_{t},t,\emptyset )) \end{array}$$ (8)

$c_{struc}=\{m,x_{0}⊙(1-m),s\}$ denotes the complete condition containing structural information; ∅ represents the empty conditional input; and $\omega_{struc}\geq 1$ is the structural guidance scale, which is set to $\omega_{struc}=2.5$ in this study. This mechanism enhances the model’s ability to constrain pattern contours, crack orientations, and edge structures, thereby preventing the restoration results from deviating from the original geometric structure of the artifact.

Overall, the data flow of the Structure-Guided Diffusion Inpainting Module consists of “damage mask and structural feature input–forward noise diffusion–conditional denoising restoration–structure-guided sampling output”. The mask generated by the detection module determines the regions to be restored, the structural feature map constrains texture and boundary continuity, and the diffusion model progressively recovers the missing content through iterative denoising. With these mechanisms, the model can achieve automated restoration with reasonable structure, natural texture, and visual continuity in regions affected by complex cracks, local defects, and damaged decorative patterns.

### CLIP-IQA for quality assessment and feedback

**Fig 4:**
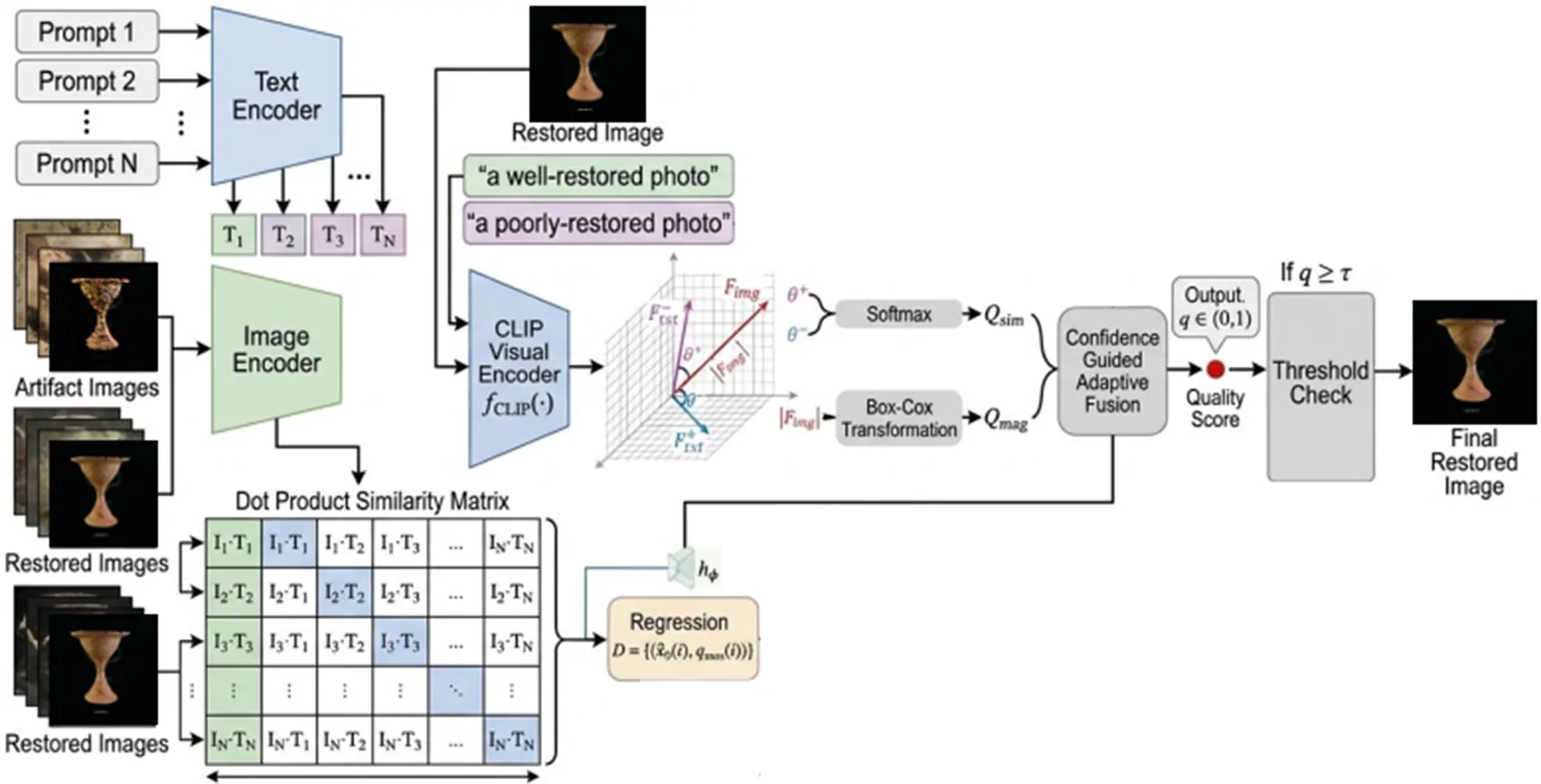
CLIP-based iterative quality assessment and feedback optimization process for adaptive restoration refinement. The research design and evaluation are based on the open-access CHVD, HBDD, and DUA datasets (see Data Availability Statement for URLs).

The CLIP-based Quality Regression and Feedback Module is the final key component of the YOLO-WSR system, and is mainly used for quality assessment and iterative control of the initial restored image generated by the Structure-Guided Diffusion Inpainting Module. This module adopts CLIP-based Image Quality Assessment (CLIP-IQA), a CLIP-based no-reference image quality assessment network, which feeds the restored image into the visual encoder to extract perceptual features and outputs a quality score ranging from 0 to 1 through a lightweight quality regression head. This score is not only used to determine whether the current restoration result satisfies the output requirement, but is also transmitted back to the Structure-Guided Diffusion Inpainting Module as a feedback signal to dynamically adjust the structural guidance strength or resample the noise seed. In this way, a closed-loop workflow integrating detection, restoration, evaluation, and feedback optimization is established. The structure is shown in Fig 4.

Let the initial restored image output by the Structure-Guided Diffusion Inpainting Module be ${\hat{x}}_{0}$. Its global semantic features are first extracted by the CLIP visual encoder $f_{CLIP}(\cdot )$, and then fed into a lightweight quality regression head $h_{\phi }(\cdot )$ to obtain the perceptual quality score. Here, $f_{CLIP}({\hat{x}}_{0})\in {\mathbb{R}}^{d}$ denotes the feature vector produced by the CLIP visual encoder, where *d* is the feature dimension. When the ViT-B/32 architecture is adopted, *d* = 512. The function $h_{\phi }:{\mathbb{R}}^{d}\to \mathbb{R}$ denotes the learnable quality regression head with parameters ϕ. σ(·) is the Sigmoid function, which compresses the output into the interval (0,1). where $s_{q}$ represents the predicted restoration quality score generated by the CLIP-based quality regression module. A value closer to 1 indicates that the restored image achieves better performance in terms of texture naturalness, structural continuity, and detail clarity. During training, the CLIP visual encoder is frozen, and only the quality regression head is trained, so as to fully exploit the pretrained semantic priors while reducing the training cost:


$$\begin{array}{c} s_{q}=\sigma (h_{\phi }(f_{CLIP}({\hat{x}}_{0}))) \end{array}$$ (9)

To enable the quality regression head to learn a restoration quality metric consistent with human perception, this study constructs a dataset of restored images and their subjective quality scores, denoted as $D=\{({\hat{x}}_{0}^{\left(i\right)},q_{mos}^{\left(i\right)}){\}}_{i=1}^{N}$. Here, $q_{mos}^{\left(i\right)}$ represents the mean subjective score corresponding to the *i*-th restored image, which is normalized to the range [0,1]. *N* denotes the total number of training samples, $q^{\left(i\right)}=\sigma (h_{\phi }(f_{CLIP}({\hat{x}}_{0}^{\left(i\right)})))$ is the quality score predicted by the model for the *i*-th restored image, and $q_{mos}^{\left(i\right)}$ is the corresponding ground-truth subjective score. With this loss function, the regression head can learn the mapping from CLIP visual features to perceptual quality scores, enabling the system to assess restoration quality under no-reference conditions:


$$\begin{array}{c} L_{reg}=\frac{1}{N}\sum_{i=1}^{N}(s_{q}^{\left(i\right)}-s_{mos}^{\left(i\right)}{)}^{2} \end{array}$$ (10)

In the inference stage, the system compares the predicted quality score $s_{q}$ with the predefined quality threshold τ. If $s_{q}\geq \tau$, the current restoration result is considered to satisfy the output requirement and no additional optimization is required. If $s_{q}<\tau$, the quality score is transformed into a quality-aware feedback coefficient and transmitted to the Structure-Guided Diffusion Inpainting Module to adjust the structural guidance scale $\omega_{struc}$. Different from directly using the quality score as the update magnitude, the proposed feedback mechanism measures the deviation between the current restoration result and the desired quality state. A lower quality score indicates larger restoration deviation and activates stronger optimization adjustment, while a higher quality score gradually reduces unnecessary refinement. Here, *k* denotes the current iteration number, $\omega_{struc}^{\left(0\right)}=2.5$ is the initial structural guidance scale, Δω=0.5 is the basic adjustment step size, and λ=5 is the sensitivity parameter controlling the influence of quality deviation on feedback intensity. To prevent excessive guidance that may introduce rigid textures or structural distortion, the guidance scale is constrained within the range of $\omega_{struc}\leq \omega_{\max}=5.0$, and the maximum number of optimization iterations is set to $K_{\max}=3$:


$$\begin{array}{c} \omega_{struc}^{\left(k+1\right)}=\min\left(\omega_{\max},\omega_{struc}^{\left(k\right)}+\Delta \omega \cdot \exp(\lambda (1-s_{q}))\right) \end{array}$$ (11)

When the quality score reaches the predefined threshold or the number of iterations reaches the upper limit, the system outputs the final restored image. Otherwise, it continues to adjust the structural guidance strength and resamples the generated result. Through this closed-loop mechanism, YOLO-WSR integrates restoration quality assessment with the generation process, enabling the automated restoration procedure to achieve not only no-reference evaluation but also adaptive optimization. As a result, the stability, controllability, and visual reliability of restored intangible cultural heritage artifact images are further improved.

## Experiment

### Datasets

In this study, three public datasets were adopted to construct the data foundation for intangible cultural heritage artifact image recognition and automated restoration tasks, namely the Cultural Heritage Visual Dataset (CHVD), DAMAGED AND UNDAMAGED ARTWORKS (DUA), and the Heritage Building Defect Detection Dataset (HBDD). For ease of description, these datasets are abbreviated as CHVD, DUA, and HBDD, respectively. Table 1 presents the basic characteristics of the three datasets and their roles in this study. CHVD is mainly used for artifact image recognition and classification tasks, DUA is primarily employed for damaged image restoration tasks, and HBDD contains defect information related to cultural heritage buildings, making it suitable for joint detection and restoration experiments.

**Table 1:** Characteristics, task roles, and experimental utilization of the datasets used in YOLO-WSR evaluation.

Dataset	Type	Main Task	Total Samples	Data Split
CHVD [34]	Cultural heritage and relic images	Cultural relic image recognition and classification	2,438 images	Train/Val/Test: 70%/15%/15%
DUA [35]	Damaged and undamaged artwork images	Cultural relic image restoration and damage recovery	116 paired restoration image groups	Train/Val/Test: 70%/15%/15%
HBDD [36]	Cultural heritage building defect images	Joint damage detection and restoration validation	1,526 images	Train/Val/Test: 70%/15%/15%

In the CHVD dataset, this study mainly selected samples related to cultural heritage artifacts, traditional artworks, historical architectural components, and heritage images, while removing samples with weak relevance to the research objective, poor visual quality, severe distortion, or unclear subjects. After data filtering, a total of 2,438 valid images were retained and organized according to their original category annotations to construct the artifact recognition dataset. For the recognition and detection tasks, all images were resized to the required input resolution of the model and normalized using consistent preprocessing settings. To improve robustness against variations in illumination, viewpoints, and artifact materials, data augmentation strategies including random horizontal flipping, brightness perturbation, contrast adjustment, color transformation, and slight rotation were applied during training. The processed images and corresponding labels were then used for training and evaluating the Wavelet Feature Upsampling Detection Module.

In the DUA dataset, this study selected 116 paired damaged and undamaged artwork image groups containing representative degradation patterns, including breakage, cracks, fading, missing regions, and surface contamination. The intact artwork images were regarded as restoration references, while the corresponding damaged images were used as restoration inputs. Since restoration evaluation requires spatial supervision information, damage masks were constructed for each damaged sample according to the visible damaged regions. Specifically, the initial damage areas were manually annotated based on the damaged regions in the images, followed by morphological refinement to generate smoother binary restoration masks. In addition, structural feature maps, such as edge representations, were extracted from the input images and jointly used with damage masks as conditional guidance for the Structure-Guided Diffusion Inpainting Module.

In the HBDD dataset, this study used 1,526 valid images as an additional validation benchmark to evaluate the generalization capability of YOLO-WSR in heritage-related damage detection and restoration scenarios. The dataset contains various visual defects in heritage buildings, including surface cracks, spalling, breakage, and structural deterioration. Images with clear defect regions and sufficient contextual information were retained, while samples with ambiguous defect locations or low visual quality were removed. The defect annotations provided in the dataset were used for training and evaluating the detection component, while the corresponding defect regions were converted into restoration masks to validate the complete workflow from damage localization to automated restoration.

For all three datasets, the data were divided into training, validation, and testing subsets according to the predefined experimental protocol, with the same partition strategy consistently applied in comparative experiments and ablation studies. This unified data organization ensures the fairness of evaluation and improves the reproducibility of the experimental results.

### Experimental details

To ensure the stability and reproducibility of the experimental results, all experiments in this study were conducted under a unified computational environment. The experimental platform was equipped with an Intel Xeon Gold 6330 processor, 128 GB of memory, and two NVIDIA RTX 4090 GPUs with 24 GB memory. The operating system was Ubuntu 20.04 LTS, the deep learning framework was PyTorch 2.2.0, and the CUDA version was 12.1. During model training, the Wavelet Feature Upsampling Detection Module, the Structure-Guided Diffusion Inpainting Module, and the CLIP-based Quality Regression and Feedback Module were trained separately, and the complete workflow was validated in the joint experimental stage. The input images were uniformly resized to 640×640 for the detection task and 512×512 for the restoration task to balance detail preservation and computational efficiency. All experiments were conducted using the same dataset partition strategy with a fixed random seed, and the training/validation/test sets were divided according to the ratio of 70%/15%/15% to ensure consistent evaluation.

In terms of data preprocessing, all datasets were first processed by removing low-quality, duplicated, and visually ambiguous samples. According to the requirements of different tasks, corresponding category labels, damage annotations, restoration masks, and paired restoration samples were constructed. For artifact recognition and damage detection tasks, all images were resized to the unified input resolution and normalized. Data augmentation strategies, including random flipping, scaling, brightness perturbation, color jittering, and slight rotation, were applied during training to improve robustness against variations in illumination, viewpoints, and material appearance. For the automated restoration task, damaged regions were converted into binary masks, where damaged areas were assigned as restoration regions and intact areas were retained as contextual references. The restoration masks were generated according to the annotated damage regions, followed by necessary refinement operations to obtain accurate restoration areas. Based on these masks, structural information such as edge feature maps was extracted and used as additional conditional guidance for the restoration generation process. The combination of input images, damage masks, and structural features enables the restoration module to perform structure-aware texture reconstruction rather than unconstrained image generation. Regarding data partitioning, all datasets were divided into training, validation, and testing subsets following the original organization of public datasets or predefined experimental protocols. The same partition strategy was maintained for all comparative experiments and ablation studies to ensure consistent evaluation conditions. During model optimization, the artifact damage detection module was trained using the AdamW optimizer with an initial learning rate of $1\times 10^{-4}$ and a batch size of 16. The automated restoration generation module adopted a lower learning rate of $5\times 10^{-5}$ with a batch size of 8 to ensure stable optimization. For the restoration quality assessment module, the CLIP visual encoder was frozen, and only the lightweight quality regression head was optimized. All modules adopted cosine annealing learning rate scheduling and an early stopping strategy to prevent overfitting and improve training stability.

### Evaluation metrics

For image restoration evaluation, PSNR, SSIM, LPIPS, FID, and KID are adopted to provide a comprehensive assessment from pixel-level, structural, perceptual, and distributional perspectives. Specifically, the restoration experiments are conducted based on paired damaged-undamaged cultural heritage image data, where intact images are used as ground-truth references and corresponding damaged images with annotated or generated damage regions are used as restoration inputs. Therefore, PSNR and SSIM can be reasonably applied to quantitatively compare the restored results with reference images, where PSNR measures pixel-level reconstruction fidelity and SSIM evaluates structural consistency. Meanwhile, LPIPS is introduced to measure deep perceptual differences between restored images and ground-truth images. Furthermore, FID and KID are employed to evaluate the distribution similarity between generated restoration results and reference images in the feature space. FID measures the feature distribution distance between two image sets, while KID provides a kernel-based estimation of distribution discrepancy with reduced bias. The combination of these metrics enables a more comprehensive evaluation of restoration quality, covering reconstruction accuracy, structural preservation, perceptual similarity, and generated image distribution consistency [37,38].

For the evaluation of the CLIP-IQA-based quality assessment module, MOS and SRCC are adopted to measure the consistency between automated quality prediction and human visual perception. MOS is obtained by averaging subjective quality scores provided by human evaluators under a blind evaluation protocol, where participants independently assess the restoration quality without access to model information. SRCC is then calculated to quantify the rank correlation between CLIP-IQA predictions and human subjective evaluations. Therefore, the overall evaluation framework, including mAP@50, PSNR, SSIM, LPIPS, FID, KID, MOS, and SRCC, provides a multi-dimensional assessment of YOLO-WSR, covering detection accuracy, restoration fidelity, perceptual quality, distribution similarity, and agreement with human judgment.

mAP@50 is used to measure the detection accuracy of the Wavelet Feature Upsampling Detection Module for categories such as cracks, fading, missing regions, and fine decorative patterns. This metric calculates the average precision of each category at an IoU threshold of 0.5 and then averages the results over all categories. Here, *N* denotes the total number of target categories, including cracks, fading, missing regions, and fine decorative patterns in this study. $p_{i}(r)$ represents the Precision–Recall curve function of the *i*-th category under the threshold of IoU=0.5, and *r* denotes the recall rate. The integral term $\int_{0}^{1}p_{i}(r)\;dr$ represents the average precision of the *i*-th category. A higher mAP@50 value indicates stronger localization and recognition capability of the detection module for different damage categories:


$$\begin{array}{c} mAP@50=\frac{1}{N}\sum_{i=1}^{N}\int_{0}^{1}p_{i}(r)dr \end{array}$$ (12)

PSNR is used to measure the pixel-level error between the restored image and the ground-truth image. $I_{gt}$ denotes the original ground-truth image, $I_{restored}$ denotes the restored image, and *H* and *W* represent the image height and width, respectively. *MAX* denotes the maximum pixel value of the image, which is set to 255 for 8-bit images. The unit of PSNR is dB, and a higher PSNR value indicates a smaller pixel-level difference between the restored image and the ground-truth image:


$$\begin{array}{c} PSNR=10\cdot \log_{10}\left(\frac{MAX^{2}}{\frac{1}{HW}\sum_{i=1}^{H}\sum_{j=1}^{W}[I_{gt}(i,j)-I_{restored}(i,j){]}^{2}}\right) \end{array}$$ (13)

SSIM is used to measure the similarity between the restored image and the ground-truth image in terms of luminance, contrast, and structure, and can better reflect the structural preservation capability of the restoration result. Here, *x* and *y* denote the corresponding local image patches of the restored image and the ground-truth image, respectively. $\mu_{x}$ and $\mu_{y}$ represent their mean values, $\sigma_{x}^{2}$ and $\sigma_{y}^{2}$ represent their variances, and $\sigma_{xy}$ denotes their covariance. In addition, $C_{1}=(0.01\cdot L{)}^{2}$ and $C_{2}=(0.03\cdot L{)}^{2}$, where *L* = *MAX* = 255. The final SSIM of the whole image is obtained by averaging the SSIM values of all local image patches. A value closer to 1 indicates higher structural consistency between the restored image and the ground-truth image:


$$\begin{array}{c} SSIM(x,y)=\frac{\left(2\mu_{x}\mu_{y}+C_{1})(2\sigma_{xy}+C_{2}\right)}{\left(\mu_{x}^{2}+\mu_{y}^{2}+C_{1})(\sigma_{x}^{2}+\sigma_{y}^{2}+C_{2}\right)} \end{array}$$ (14)

LPIPS is used to evaluate the difference between the restored image and the ground-truth image in the deep perceptual feature space, and can reflect texture naturalness and visual similarity from the perspective of human perception. Here, *x* and *y* denote the restored image and the ground-truth image, respectively. *l* represents the *l*-th layer of the pretrained feature extraction network, while $H_{l}$ and $W_{l}$ denote the height and width of the feature map at the *l*-th layer, respectively. ${\hat{x}}_{hw}^{l}$ and ${\hat{y}}_{hw}^{l}$ represent the feature vectors at position (*h*,*w*), $w_{l}$ denotes the learnab*l*e channel-wise weight vector, ⊙ indicates element-wise multiplication, and $\|\cdot {\|}_{2}$ represents the L2 norm. A lower LPIPS value indicates that the restored image is closer to the ground-truth image in the perceptual feature space:


$$\begin{array}{c} LPIPS(x,y)=\sum_{l}\frac{1}{H_{l}W_{l}}\sum_{h=1}^{H_{l}}\sum_{w=1}^{W_{l}}{\left\|w_{l}⊙\left(\frac{{\hat{y}}_{hw}^{l}}{\|{\hat{y}}_{hw}^{l}{\|}_{2}}-\frac{{\hat{x}}_{hw}^{l}}{\|{\hat{x}}_{hw}^{l}{\|}_{2}}\right)\right\|}_{2}^{2} \end{array}$$ (15)

SRCC is used to measure the ranking consistency between the quality scores predicted by CLIP-IQA and human subjective ratings, reflecting whether the model-predicted scores are consistent with human judgment of restoration quality. Here, *N* denotes the total number of evaluation samples, $d_{i}$ represents the rank difference of the *i*-th image between the two rankings, $q_{i}$ denotes the quality score predicted by CLIP-IQA, and $MOS_{i}$ represents the human subjective score. The value range of SRCC is [−1,1]. A value closer to 1 indicates stronger monotonic consistency between the model-predicted scores and human evaluations:


$$\begin{array}{c} \rho_{s}=1-\frac{6\sum_{i=1}^{N}d_{i}^{2}}{N(N^{2}-1)} \end{array}$$ (16)

FID is adopted to evaluate the distribution similarity between the restored images and the reference images in the deep feature space. Specifically, the feature representations of the restored image set and the ground-truth image set are extracted using the Inception network, and the statistical distributions of the two feature sets are modeled as multivariate Gaussian distributions. The FID metric measures the distance between these two distributions, where $\mu_{r}$ and $\mu_{g}$ denote the mean vectors of the reference and restored image features, respectively. $\Sigma_{r}$ and $\Sigma_{g}$ represent the corresponding covariance matrices, and Tr(·) denotes the trace operation. A lower FID value indicates that the distribution of the restored images is closer to that of the reference images, reflecting better visual realism and feature-level consistency:


$$\begin{array}{c} FID=\|\mu_{r}-\mu_{g}{\|}_{2}^{2}+Tr(\Sigma_{r}+\Sigma_{g}-2(\Sigma_{r}\Sigma_{g}{)}^{\frac{1}{2}}) \end{array}$$ (17)

KID is introduced as a complementary metric to measure the distribution discrepancy between restored images and reference images through kernel-based feature distribution comparison. Unlike FID, KID provides an unbiased estimation of the maximum mean discrepancy (MMD) between two feature distributions. Given feature representations $\{x_{i}{\}}_{i=1}^{m}$ and $\{y_{j}{\}}_{j=1}^{n}$ extracted from restored and reference images, respectively, k(·,·) denotes the polynomial kernel function. The KID value reflects the difference between the two image distributions in the feature space, where a lower value indicates that the generated restoration results have a distribution closer to the real reference images:


$$\begin{array}{c} KID=\frac{1}{m(m-1)}\sum_{i=\;\;/j}k(x_{i},x_{j})+\frac{1}{n(n-1)}\sum_{i=\;\;/j}k(y_{i},y_{j})-\frac{2}{mn}\sum_{i=1}^{m}\sum_{j=1}^{n}k(x_{i},y_{j}) \end{array}$$ (18)

Through the above seven metrics, this study comprehensively evaluates YOLO-WSR from multiple perspectives, including detection accuracy, pixel-level restoration fidelity, structural consistency, perceptual quality, distribution similarity, and consistency with human visual perception. This evaluation system provides comprehensive coverage of the detection, restoration generation, and feedback optimization processes of the proposed framework, and establishes a unified and reliable quantitative basis for subsequent comparative experiments and ablation studies.

### Comparative experiments and analysis

To comprehensively validate the overall performance of YOLO-WSR in cultural heritage artifact image recognition, automated restoration, and quality feedback optimization associated with intangible cultural heritage preservation, comparative experiments were conducted on three public datasets, namely CHVD, DUA, and HBDD. The evaluation metrics cover five aspects, including object detection accuracy, pixel-level restoration fidelity, structural consistency, perceptual quality, and quality assessment consistency, thereby providing a comprehensive evaluation of the model performance across detection, restoration generation, and feedback optimization stages. WPSW-YOLOv8, MS-YOLOv8-DBHead, and DKR-YOLO were selected as comparative detection models, while K2Mural, GuidePaint, and MANIQA were used as comparative models for restoration and quality assessment. Under unified data processing protocols and evaluation metrics, a multi-dimensional performance analysis of YOLO-WSR was conducted, as shown in Tables 2 and 3.

**Table 2:** Performance comparison of YOLO-WSR and baseline methods on cultural heritage artifact damage detection.

Model	Dataset	mAP@50
WPSW-YOLOv8 [39]	CHVD	86.3 ± 0.8
	HBDD	84.7 ± 0.9
MS-YOLOv8-DBHead [40]	CHVD	86.6 ± 0.7
	HBDD	85.2 ± 0.8
DKR-YOLO [41]	CHVD	87.1 ± 0.6
	HBDD	85.8 ± 0.7
YOLO-WSR	CHVD	91.6 ± 0.5
	HBDD	90.4 ± 0.5

**Table 3:** Performance comparison of YOLO-WSR and baseline methods on artifact image restoration, perceptual quality assessment, and distribution similarity evaluation.

Model	Dataset	PSNR	SSIM	LPIPS	SRCC	FID	KID
K2Mural [42]	DUA	28.3±0.4	0.835±0.007	–	–	48.6±1.8	0.041±0.003
	HBDD	27.8±0.5	0.823±0.008	–	–	52.1±2.0	0.045±0.003
GuidePaint [43]	DUA	29.0±0.4	0.848±0.006	0.150±0.005	–	42.3±1.6	0.035±0.002
	HBDD	28.5±0.4	0.838±0.007	0.157±0.006	–	46.5±1.7	0.039±0.003
MANIQA [44]	DUA	29.6±0.3	0.862±0.005	0.138±0.004	0.790±0.012	39.8±1.5	0.031±0.002
	HBDD	29.1±0.4	0.851±0.006	0.145±0.005	0.781±0.011	43.2±1.6	0.034±0.002
LaMa [45]	DUA	30.2±0.3	0.874±0.005	0.125±0.004	0.810±0.010	35.7±1.4	0.027±0.002
	HBDD	29.7±0.4	0.860±0.006	0.130±0.005	0.803±0.011	38.9±1.5	0.030±0.002
ZITS [46]	DUA	30.5±0.3	0.880±0.004	0.120±0.003	0.825±0.010	33.4±1.3	0.025±0.002
	HBDD	30.0±0.4	0.865±0.005	0.125±0.004	0.815±0.011	36.7±1.4	0.028±0.002
YOLO-WSR	DUA	32.2±0.3	0.916±0.004	0.086±0.003	0.891±0.010	24.8±1.1	0.016±0.001
	HBDD	31.5±0.4	0.903±0.005	0.092±0.003	0.879±0.011	27.6±1.2	0.019±0.001

To ensure a fair comparison, all baseline methods were carefully adjusted before experimental evaluation to maintain consistent experimental conditions with the proposed YOLO-WSR framework. Specifically, all restoration-related baselines were evaluated using the same test images, damage masks, image resolutions, and preprocessing procedures. For methods originally designed for general image restoration or inpainting, their input formats were modified according to the requirements of cultural heritage artifact image restoration, while keeping their original network structures and optimization principles unchanged. The output images of all restoration methods were generated under the same damage-region constraints and were subsequently evaluated using identical quantitative metrics, including PSNR, SSIM, LPIPS, FID, KID, and SRCC. For image quality assessment methods, such as MANIQA, which are originally designed for no-reference image quality evaluation rather than restoration generation, the model was not considered as a direct restoration baseline. Instead, it was adjusted to operate as an auxiliary quality evaluation method by providing quality prediction results for the restored images under the same test settings. In addition, all baseline models were configured with the same input image size and evaluated using identical test protocols to reduce the influence of implementation differences. These adjustments ensure that the comparison reflects the actual performance differences among methods under consistent experimental conditions.

**Fig 5:**
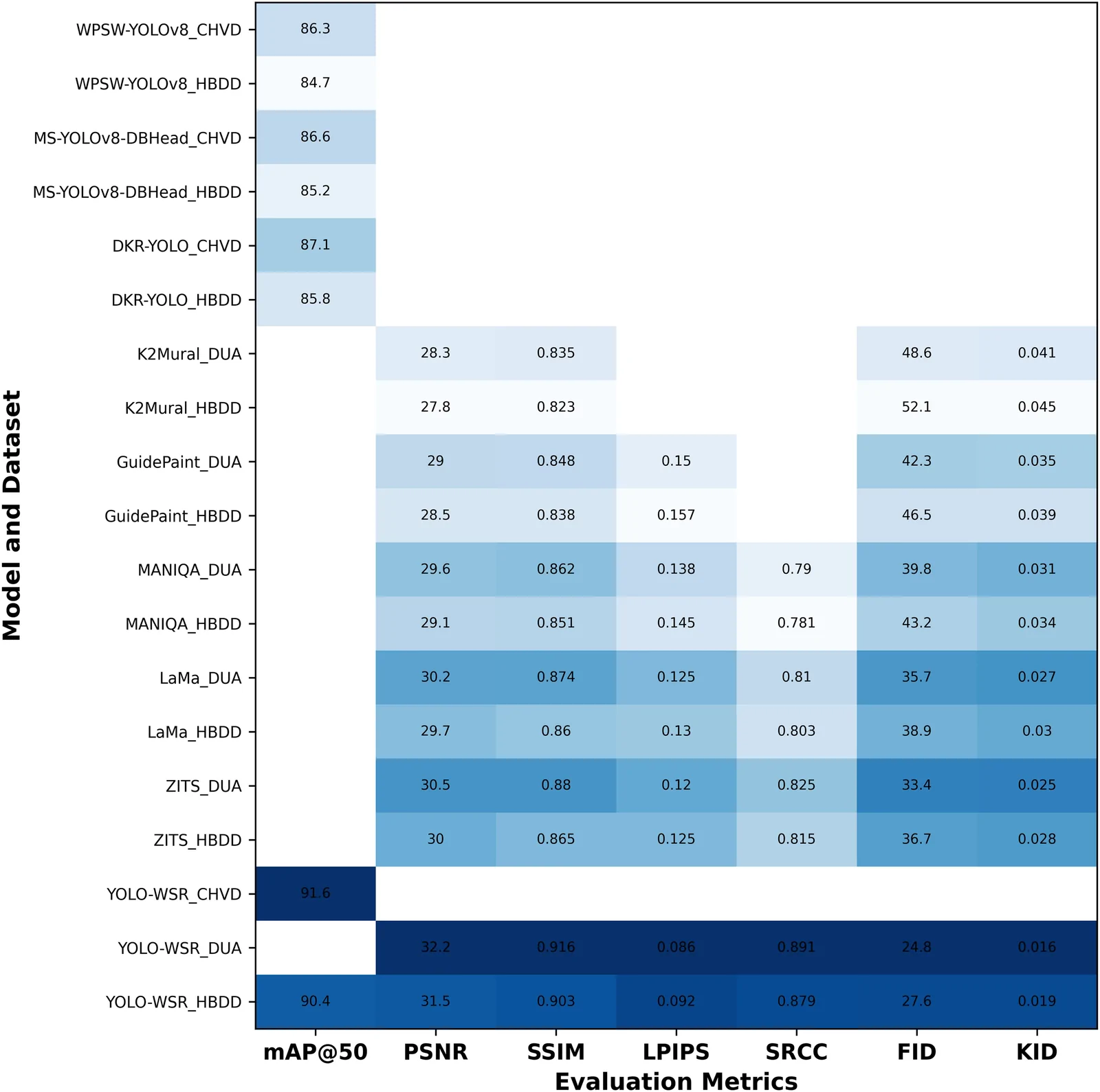
Quantitative comparison of YOLO-WSR and baseline methods on detection and restoration evaluation metrics.

As shown in Fig 5, YOLO-WSR achieves consistent improvements over the comparison methods in the object detection task. On the CHVD dataset, YOLO-WSR improves mAP@50 by approximately 5%–6% compared with the three YOLO-based baselines. This improvement indicates that the proposed framework provides more accurate localization and recognition of artifact damage categories, including cracks, fading regions, missing areas, and fine decorative patterns. In complex backgrounds and texture-rich scenes, YOLO-WSR can better capture subtle local structures and high-frequency details, which benefits from the WFU-based feature enhancement strategy. By improving multi-scale feature representation, the detection module generates more informative damage masks and feature maps, providing more reliable spatial guidance for the subsequent restoration stage. On the HBDD dataset, YOLO-WSR also maintains stable detection advantages, achieving approximately 5%–7% higher mAP@50 than the compared methods. These results suggest that wavelet-enhanced feature representation improves the perception capability of small-scale and irregular defects, especially for heritage images with diverse textures and complex boundaries. Overall, the detection results demonstrate that YOLO-WSR provides more effective damage localization, which establishes a better information foundation for the following restoration process.

YOLO-WSR also demonstrates improved performance in artifact image restoration and perceptual quality evaluation. On the DUA dataset, YOLO-WSR achieves higher PSNR and SSIM values than the comparison methods, indicating improved pixel-level reconstruction accuracy and structural consistency. Meanwhile, the lower LPIPS value suggests that the restored images have reduced perceptual distance from the reference images, reflecting better visual feature consistency. Compared with traditional restoration approaches such as K2Mural and GuidePaint, YOLO-WSR produces more continuous textures and more coherent structures in damaged regions, particularly for complex cracks, missing areas, and fine decorative patterns. Compared with MANIQA, LaMa, and ZITS, YOLO-WSR further improves restoration fidelity and perceptual consistency, which indicates the effectiveness of integrating detection-guided spatial priors and structure-guided diffusion reconstruction. In addition, the higher SRCC value demonstrates that the quality evaluation results generated by YOLO-WSR are more consistent with human perceptual judgments. The newly introduced FID and KID metrics further show that YOLO-WSR achieves closer feature distributions to the reference images, suggesting improved distributional similarity and perceptual realism of the generated restoration results.

On the HBDD dataset, YOLO-WSR maintains similar advantages under more complex heritage building defect scenarios. Compared with the baseline restoration methods, YOLO-WSR achieves improvements in PSNR and SSIM while reducing LPIPS, indicating enhanced reconstruction quality and lower perceptual discrepancy. The results show that the proposed structure-guided diffusion restoration module can better preserve spatial continuity and recover texture details in regions containing cracks, spalling, and structural defects. Furthermore, the improvements in SRCC, FID, and KID suggest that the restored images are not only closer to reference distributions but also better aligned with perceptual quality evaluation. These results indicate that the combination of detection-guided restoration, structural constraints, and CLIP-based feedback optimization contributes to more adaptive and consistent restoration performance.

The computational efficiency of YOLO-WSR was further evaluated through three repeated experiments on the CHVD, DUA, and HBDD datasets. As shown in Table 4, the proposed framework maintains consistent computational characteristics across different experiments and datasets. The variation of parameter count and FLOPs among different runs remains within a very small range, indicating that the model structure and computational complexity are stable during repeated evaluations. In terms of inference efficiency, the maximum fluctuation of inference time among repeated experiments is less than 3%, while the difference among different datasets remains within approximately 12%. This slight variation is mainly related to the complexity of image contents and damage structures in different datasets. Although YOLO-WSR integrates multiple functional components, including wavelet-enhanced damage detection, diffusion-based restoration, and CLIP-based quality feedback optimization, the computational analysis indicates that the framework maintains stable resource consumption while achieving improved restoration performance. These results suggest that the proposed framework provides a reasonable balance between restoration effectiveness and computational efficiency, supporting its potential application in practical cultural heritage image processing scenarios.

**Table 4:** Computational efficiency analysis of YOLO-WSR in terms of parameter size, computational complexity, and inference speed under repeated experiments.

Experiment	Dataset	Parameters (M)	FLOPs (G)	Inference Time (ms)
Run 1	CHVD	38.42	85.9	48.6
	DUA	38.51	86.2	51.4
	HBDD	38.63	86.7	54.2
Run 2	CHVD	38.45	86.0	49.1
	DUA	38.55	86.3	50.7
	HBDD	38.68	86.8	53.6
Run 3	CHVD	38.39	85.8	48.2
	DUA	38.48	86.1	51.0
	HBDD	38.60	86.6	54.5

**Fig 6:**
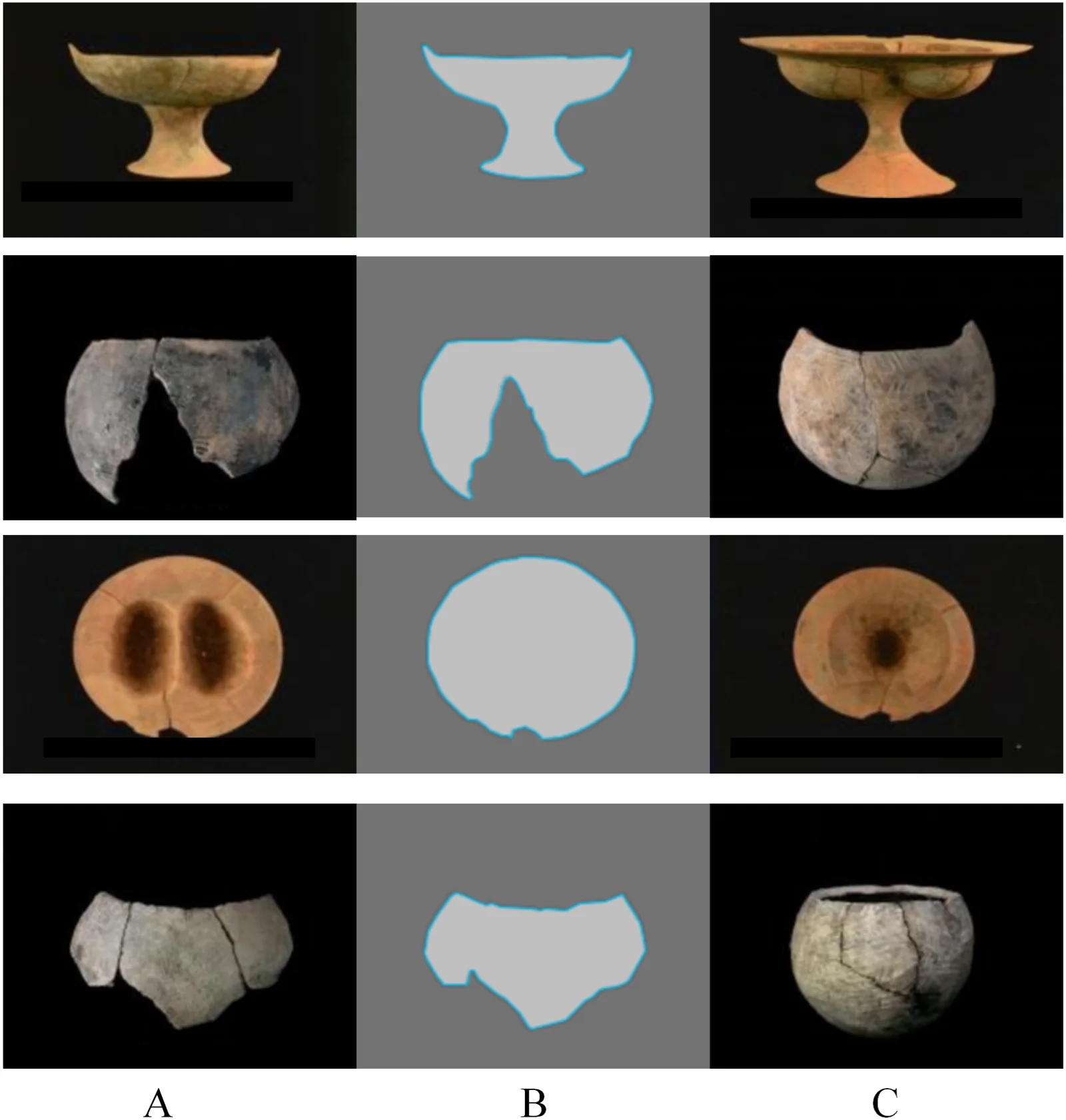
Qualitative visualization of YOLO-WSR detection and restoration results. (A) Original artifact images, (B) detected damage regions and masks, and (C) corresponding restored images.The research design and evaluation are based on the open-access CHVD, HBDD, and DUA datasets (see Data Availability Statement for URLs).

In the object detection task, YOLO-WSR achieves consistent improvements on the CHVD dataset compared with the three comparative models, with an increase of approximately 5%–6% in mAP@50. Meanwhile, the smaller performance variation indicates that YOLO-WSR maintains stable detection capability across different image samples. These results suggest that the proposed wavelet-enhanced detection module improves the recognition of damage categories, including cracks, fading regions, missing areas, and fine decorative patterns. On the HBDD dataset, YOLO-WSR also achieves approximately 5%–6% higher mAP@50 than the comparison methods, indicating improved robustness when handling complex textures, irregular defects, and diverse architectural damage patterns. In the restoration task, YOLO-WSR achieves higher PSNR and SSIM values on the DUA dataset, demonstrating improved pixel-level reconstruction accuracy and structural consistency. In addition, the lower LPIPS value indicates reduced perceptual distance between the restored images and reference images, while the higher SRCC value suggests better consistency between automated quality assessment and human perceptual evaluation. On the HBDD dataset, YOLO-WSR maintains similar advantages, achieving improvements in reconstruction fidelity, structural preservation, and perceptual quality evaluation. The reduced LPIPS value and improved SRCC results indicate that the proposed framework can better preserve texture continuity and structural information in complex heritage damage scenarios. Overall, the experimental results demonstrate that YOLO-WSR provides stable performance across different datasets by integrating detection-guided restoration, structure-constrained diffusion generation, and CLIP-based quality feedback optimization. The observed improvements suggest that the proposed closed-loop design contributes to more consistent and adaptive cultural heritage artifact image restoration under the evaluated experimental settings. Fig 6 presents the detection and restoration results of YOLO-WSR.

YOLO-WSR demonstrates improved performance in both object detection and image restoration tasks, achieving consistent gains across multiple evaluation metrics. In terms of detection accuracy, pixel-level fidelity, structural consistency, perceptual quality, and consistency with subjective quality scores, YOLO-WSR outperforms the comparative models under the evaluated experimental settings. Moreover, the smaller performance variations across different samples and datasets indicate that the proposed framework maintains stable and consistent performance. Overall, the experimental results suggest that YOLO-WSR provides an effective approach for end-to-end recognition and restoration of cultural heritage artifact images associated with intangible cultural heritage preservation, demonstrating its potential value for intelligent cultural heritage image processing applications.

### Ablation experiments and analysis

To further investigate the contribution of each core module and internal component in YOLO-WSR to the overall performance, this study designed comprehensive ablation experiments [47]. Specifically, two levels of ablation variants were constructed. First, the complete modules were removed sequentially, including the Wavelet Feature Upsampling Detection Module (w/o WFD), the Structure-Guided Diffusion Inpainting Module (w/o SGDI), and the CLIP-based Quality Regression and Feedback Module (w/o CQRF), to analyze the contribution of each major component. Furthermore, to provide a more detailed analysis of the internal mechanisms, additional ablation variants were introduced, including w/o WFU, w/o Diffusion Restoration, and w/o CLIP. All variants were evaluated on the corresponding datasets, and the experimental results are presented in Table 5.

**Table 5:** Ablation study of individual components in the YOLO-WSR framework and their contributions to detection and restoration performance.

Model	Dataset	mAP@50	PSNR	SSIM	LPIPS	SRCC
w/o WFD	CHVD	86.8	31.5	0.892	0.098	0.875
	DUA	88.2	31.8	0.897	0.096	0.879
	HBDD	87.3	31.1	0.887	0.101	0.869
w/o WFU	CHVD	90.9	32.0	0.910	0.090	0.884
	DUA	91.1	32.3	0.914	0.087	0.889
	HBDD	90.1	31.5	0.902	0.093	0.874
w/o SGDI	CHVD	90.5	29.6	0.871	0.120	0.850
	DUA	90.8	29.9	0.875	0.116	0.853
	HBDD	89.9	29.4	0.869	0.123	0.846
w/o Diffusion Restoration	CHVD	91.0	29.8	0.876	0.117	0.854
	DUA	91.2	30.1	0.881	0.113	0.858
	HBDD	90.2	29.6	0.873	0.119	0.851
w/o CQRF	CHVD	91.1	32.0	0.912	0.088	0.884
	DUA	91.3	32.2	0.915	0.086	0.887
	HBDD	90.2	31.4	0.902	0.092	0.872
w/o CLIP Feedback	CHVD	91.2	32.1	0.914	0.087	0.887
	DUA	91.4	32.4	0.917	0.084	0.891
	HBDD	90.3	31.6	0.905	0.090	0.876
YOLO-WSR	CHVD	91.6	32.3	0.917	0.085	0.892
	DUA	91.8	32.6	0.920	0.083	0.895
	HBDD	90.5	31.8	0.908	0.089	0.881

In the ablation experiments, to ensure experimental feasibility and continuity of the data flow, the removed modules were replaced with corresponding placeholder or default operations, so as to avoid interruptions in the overall network architecture. When WFD was removed, the restoration module directly used the original input image and a pre-generated unified mask as inputs to maintain consistency in input dimensions and data format. When SGDI was removed, the quality assessment module received an initial restored image generated by simple texture filling, ensuring that the input features remained available for computation. When CQRF was removed, the generation module adopted a fixed structural guidance strength for texture completion and iterative sampling, thereby maintaining the continuity of the restoration process. Through this replacement strategy, the contribution of each module could be independently evaluated in the ablation experiments, while the data flow and training process of different network components remained unaffected, leading to reliable analysis of performance degradation.

The ablation results demonstrate that each component in YOLO-WSR is designed to address a specific challenge in cultural heritage artifact image processing and contributes to different aspects of the overall performance. The YOLO-based detection module with Wavelet Feature Upsampling (WFU) is introduced to overcome the difficulty of accurately locating small-scale, irregular, and texture-sensitive damage regions. By enhancing high-frequency details and multi-scale feature representations in the wavelet domain, WFU improves the detection capability for subtle cracks, fading areas, missing regions, and fine decorative patterns. After removing the WFU strategy, the detection performance decreases notably, with the mAP@50 reduced by approximately 0.8%–1.0% across different datasets. Since the detection results are further used as spatial and texture priors for restoration, the absence of WFU also leads to a slight degradation in restoration performance, with PSNR and SSIM decreasing by approximately 1%–2% and LPIPS increasing by about 10%–15%. These results indicate that WFU does not simply increase model complexity, but provides more discriminative feature representations for accurate damage localization and subsequent restoration. Meanwhile, the Structure-Guided Diffusion Restoration component is designed to solve the problems of incomplete texture recovery and structural inconsistency during restoration. By incorporating damage masks and structural constraints into the diffusion generation process, this component guides missing-region reconstruction and preserves continuity between damaged and intact areas. When the diffusion restoration component is removed, PSNR and SSIM decrease by approximately 7%–9%, while LPIPS increases by more than 30% on average. The decrease in SRCC further indicates that the absence of structure-guided diffusion weakens the consistency between restoration results and perceptual quality evaluation, confirming its importance in texture reconstruction and structural preservation.

**Fig 7:**
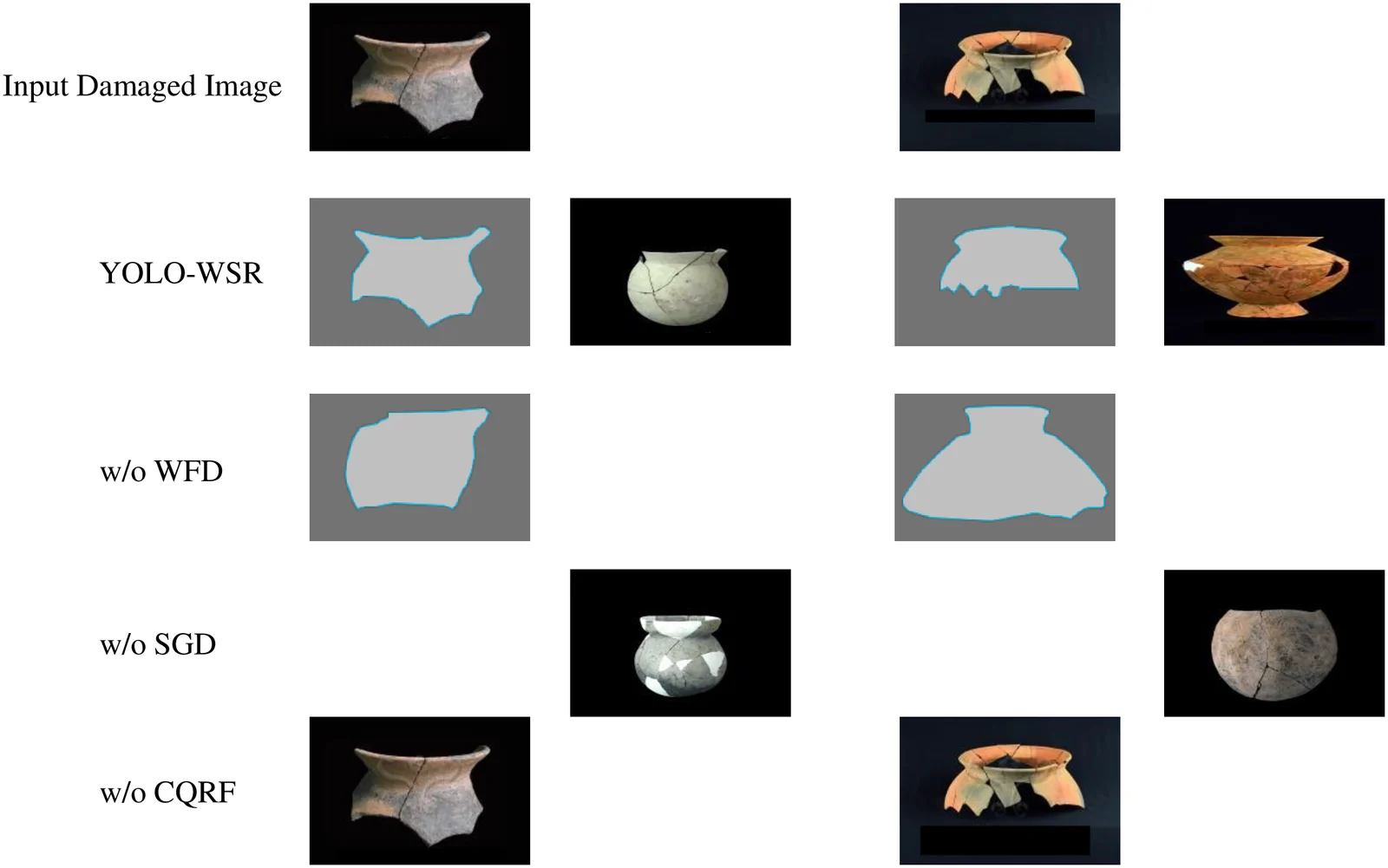
Qualitative comparison of YOLO-WSR and its ablation variants with different components removed. The first row shows the input damaged images. For each ablation variant, the left images represent the predicted damage localization results, while the right images represent the corresponding restoration results. Blank regions indicate that the corresponding function is unavailable after removing the specific module.The research design and evaluation are based on the open-access CHVD, HBDD, and DUA datasets (see Data Availability Statement for URLs).

The CQRF mechanism and CLIP-based feedback optimization are introduced to address the limitation of fixed restoration generation without adaptive quality regulation. By evaluating restoration quality and providing iterative feedback, this component dynamically adjusts the restoration process according to the quality deviation of the current output. Without CQRF, SRCC decreases by approximately 1%–3%, while LPIPS increases by about 5%–8%, indicating reduced perceptual consistency and weaker alignment with quality evaluation. Similarly, disabling the CLIP Feedback mechanism reduces the iterative refinement capability, resulting in slight decreases in PSNR and SSIM and increased perceptual discrepancy. These results demonstrate that the feedback mechanism does not merely introduce additional computational overhead, but provides quality-aware optimization after initial restoration. Overall, the ablation experiments verify that the performance improvement of YOLO-WSR originates from the coordinated interaction among wavelet-enhanced damage detection, structure-constrained diffusion restoration, and feedback-driven quality optimization, rather than from a simple accumulation of existing techniques. Fig 7 presents the experimental results of YOLO-WSR after module ablation.

However, although single-module ablation experiments can reveal the independent contribution of each module in YOLO-WSR, they cannot fully reflect the synergistic effects and interdependencies among different modules. Therefore, to further analyze the joint effects among modules, multi-module ablation experiments were designed [48]. By sequentially removing different combinations of modules from YOLO-WSR, the overall influence of different module combinations on detection accuracy, restoration quality, and quality assessment consistency can be examined in greater depth. These experiments aim to quantify the importance of module collaboration and evaluate their comprehensive role in end-to-end cultural heritage artifact image processing associated with intangible cultural heritage preservation, thereby providing evidence for understanding the mechanism behind the performance improvement of the proposed model. Table 6 presents the experimental results after multi-module ablation.

**Table 6:** Ablation results of YOLO-WSR under different module removal strategies.

Model	Dataset	mAP@50	PSNR	SSIM	LPIPS	SRCC
w/o WFD+SGDI	CHVD	85.2	29.0	0.860	0.120	0.840
	DUA	88.0	28.5	0.870	0.125	0.845
	HBDD	84.5	28.0	0.855	0.130	0.835
w/o SGDI+CQRF	CHVD	89.5	30.0	0.890	0.098	0.865
	DUA	90.0	29.2	0.875	0.110	0.855
	HBDD	89.0	29.0	0.880	0.112	0.850
w/o WFD+CQRF	CHVD	88.5	31.0	0.900	0.092	0.870
	DUA	89.2	30.5	0.895	0.095	0.875
	HBDD	87.8	30.0	0.885	0.100	0.860
YOLO-WSR	CHVD	91.6	32.2	0.916	0.086	0.891
	DUA	32.2	32.2	0.916	0.086	0.891
	HBDD	90.4	31.5	0.903	0.092	0.879

As shown in Table 5, when any two modules are removed from YOLO-WSR, the performance decreases significantly across all metrics. On the CHVD dataset, after removing WFD+SGDI, mAP@50 decreases by approximately 6%–7%, while PSNR and SSIM decrease by about 7%–8%, respectively. This indicates that the spatial and texture priors provided by the object detection output have a direct influence on subsequent restoration quality. LPIPS increases by approximately 15%, reflecting a larger perceptual gap between the generated image and the original image, while SRCC decreases by about 9%, indicating a clear reduction in the consistency of subjective quality assessment. These results verify the synergistic role of WFD and SGDI in the detection and restoration workflow on the CHVD dataset. On the DUA dataset, after removing SGDI+CQRF, PSNR decreases by approximately 8%–10%, SSIM decreases by about 7%, and LPIPS increases by more than 18%, indicating insufficient texture continuity and degraded perceptual quality. SRCC decreases by approximately 10%, showing weakened consistency between subjective scores and predicted scores. Meanwhile, mAP@50 also shows a slight decrease of about 5%, suggesting that the absence of the restoration generation and feedback optimization modules also has a certain impact on the detection task. This reflects the interdependence among modules in high-fidelity texture generation and structural continuity on the DUA dataset. On the HBDD dataset, after removing WFD+CQRF, mAP@50 decreases by approximately 5%–6%, PSNR and SSIM decrease by about 6%–8%, LPIPS increases by approximately 16%, and SRCC decreases by about 8%. These results indicate that the spatial and texture information provided by the detection module, together with feedback optimization, is essential for maintaining natural textures and structural continuity in restored images. In addition, removing SGDI and CQRF, i.e., w/o SGDI+CQRF, leads to a PSNR decrease of approximately 8%, an SSIM decrease of about 7%, an LPIPS increase of approximately 18%, and an SRCC decrease of about 10%. This demonstrates that automated restoration generation and feedback optimization play a critical role in texture completion, structural preservation, and subjective perceptual consistency for architectural cultural heritage images.

Different ablation combinations affect the metrics of YOLO-WSR to varying degrees. On the CHVD dataset, detection accuracy and structural fidelity are most strongly affected when WFD+SGDI are removed. On the DUA dataset, pixel-level restoration fidelity, structural consistency, and perceptual quality show the most pronounced degradation when SGDI+CQRF are removed. On the HBDD dataset, the decline across different metrics is relatively balanced, but the changes in LPIPS and SRCC are particularly evident, highlighting the importance of module collaboration in scenarios with complex textures and rich structural information. Among the five metrics, mAP@50 is the most sensitive to the detection task; PSNR and SSIM reflect the contributions to restoration fidelity and structural consistency; LPIPS measures perceptual similarity; and SRCC evaluates consistency with subjective quality scores. The decrease in all metrics in the multi-module ablation experiments indicates that the end-to-end performance of YOLO-WSR depends on the collaboration and complementarity among different modules. By analyzing the performance of different ablation combinations across the three datasets, the indispensable role of each module in ensuring detection accuracy, restoration quality, and perceptual consistency can be clearly understood, as well as their joint contribution within the end-to-end processing workflow.

**Fig 8:**
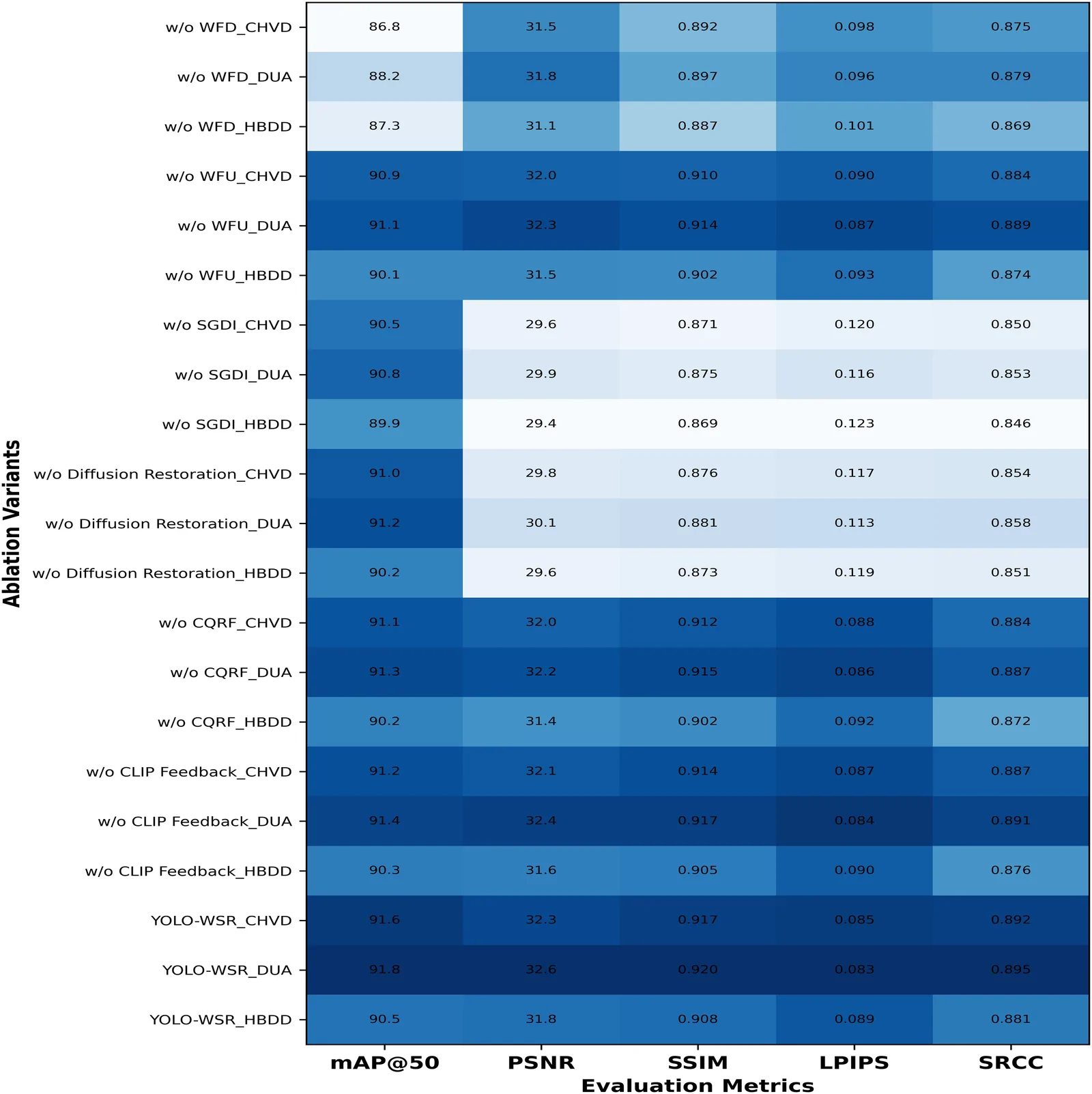
Quantitative comparison of YOLO-WSR ablation variants under different module configurations.

The results in Fig 8 indicate that there are synergistic effects among the modules of YOLO-WSR, and the absence of any module leads to performance degradation in detection accuracy, restoration fidelity, structural consistency, perceptual quality, and consistency with subjective scores. The comparison across datasets shows that different module combinations have varying effects on different task-specific metrics, while all results consistently demonstrate the importance of effective collaboration among modules. These findings indicate the rationality of the YOLO-WSR design for cultural heritage artifact image processing associated with intangible cultural heritage preservation and highlight the contribution of each component to improving the overall framework performance.

## Conclusion and discussion

This study proposes YOLO-WSR, an end-to-end framework for the recognition and automated restoration of cultural heritage artifact images associated with intangible cultural heritage preservation. The model consists of three components: the Wavelet Feature Upsampling Detection Module (WFD), the Structure-Guided Diffusion Inpainting Module (SGDI), and the CLIP-based Quality Regression and Feedback Module (CQRF). These modules jointly form a complete workflow covering accurate localization of damaged regions, texture and structural completion, quality assessment, and closed-loop optimization. Through the coordinated integration of wavelet-based feature enhancement, multi-scale information fusion, structure-guided diffusion generation, and a CLIP-IQA feedback mechanism, YOLO-WSR demonstrates improved performance in detection accuracy, restoration fidelity, structural continuity, and perceptual quality. The proposed framework provides an effective approach for the digital processing and automated restoration of cultural heritage artifact images, offering potential support for intelligent cultural heritage preservation applications.

In terms of experimental evaluation, YOLO-WSR was systematically validated across target detection, restoration generation, and quality assessment tasks. The comparative results show that YOLO-WSR consistently outperforms existing mainstream methods across multiple metrics. Specifically, the average detection accuracy is improved by approximately 5%–6%, while the pixel-level restoration fidelity, measured by PSNR, increases by about 10%. These results demonstrate the advantages of the proposed model in fine-grained texture recognition and high-fidelity restoration. Meanwhile, the ablation analysis further confirms the indispensable role and synergistic contribution of each module within the end-to-end workflow, providing direct evidence for understanding the performance improvement mechanism of YOLO-WSR and demonstrating its robustness and reliability across different tasks and application scenarios.

In future work, YOLO-WSR can be further extended and optimized from several perspectives. First, more efficient feature enhancement methods and lightweight generative strategies can be explored to better accommodate large-scale or high-resolution artifact images. Second, multimodal information, such as depth maps, infrared imaging, or historical reference images, can be incorporated to improve the recognition and restoration of complex textures and subtle damage. In addition, more intelligent quality evaluation and adaptive optimization mechanisms can be introduced to enable real-time restoration feedback, thereby further improving the consistency between restoration results and human perceptual judgment. Finally, enhancing the generalization capability of the model across different categories of cultural heritage artifacts and practical conservation scenarios represents an important direction for future research.
